# Workforce and institutional factors associated with comprehensive genomic profiling utilization in gynecologic oncology: a nationwide questionnaire survey in Japan

**DOI:** 10.1007/s10147-026-03143-7

**Published:** 2026-07-27

**Authors:** Tamotsu Sudo, Kenta Masuda, Katsutoshi Oda, Hidemichi Watari, Akira Hirasawa, Shinya Sato, Kenichi Harano, Satoru Nagase, Kazuhiro Takehara, Masaki Mandai, Yuko Sasajima, Hirouki Yanai, Hitoshi Tsuda, Yusuke Kobayashi, Kei Kawana, Mikio Mikami, Kiyoko Kato, Daisuke Aoki, Aikou Okamoto

**Affiliations:** 1 Department of Cancer Genetics and Genomics, School of Medicine, Fujita Health University, Toyoake, Aichi Japan; 2 Department of Obstetrics and Gynecology, Keio University School of Medicine, Tokyo, Japan; 3 Division of Integrative Genomics, Graduate School of Medicine, The University of Tokyo, Tokyo, Japan; 4 Department of Obstetrics and Gynecology, Graduate School of Medicine and Faculty of Medicine, Hokkaido University, Sapporo, Japan; 5 Department of Clinical Genomic Medicine, Dentistry and Pharmaceutical Sciences, Okayama University Graduate School of Medicine, Okayama, Japan; 6 Department of Obstetrics and Gynecology, Tottori University School of Medicine, 36-1 Nishicho, Yonago, Tottori, 683-8504 Japan; 7 Department of Medical Oncology, National Cancer Center Hospital East, Kashiwa, Japan; 8 Department of Obstetrics and Gynecology, Faculty of Medicine, Yamagata University, Yamagata, Japan; 9 Department of Gynecologic Oncology, NHO Shikoku Cancer Center, Matsuyama, Ehime, Japan; 10 Department of Gynecology and Obstetrics, Kyoto University Graduate School of Medicine, Kyoto, Japan; 11 Department of Pathology, Teikyo University Hospital, Tokyo, Japan; 12 Department of Pathology, Dentistry, and Pharmaceutical Sciences, Okayama University Graduate School of Medicine, Okayama, Japan; 13 Department of Basic Pathology, National Defense Medical College, Tokorozawa, Saitama Japan; 14 Department of Obstetrics and Gynecology, Institute of Medicine, University of Tsukuba, Ibaraki, Japan; 15 Department of Obstetrics and Gynecology, Nihon University School of Medicine, Tokyo, Japan; 16 Department of Medical Science, Shonan University of Medical Science, Kanagawa, Japan; 17 Department of Gynecology and Obstetrics, Graduate School of Medical Sciences, Kyushu University, Fukuoka, Japan; 18 International University of Health and Welfare Graduate School, Tokyo, Japan; 19 Department of Obstetrics and Gynecology, The Jikei University School of Medicine, Minato-Ku, Tokyo, Japan

**Keywords:** Comprehensive genomic profiling, Gynecological oncology, Precision oncology, Cancer genomic medicine, Organ-specialty–led implementation, Germline findings

## Abstract

**Background:**

Comprehensive genomic profiling (CGP) has been widely introduced into precision oncology; however, its real-world implementation in gynecologic oncology remains unclear. This study evaluated nationwide CGP utilization, treatment translation, and management of secondary germline findings in Japanese gynecologic oncology.

**Methods:**

A nationwide survey was conducted across 98 institutions participating in gynecologic oncology training and/or Japan’s cancer genomic medicine network. Institutional characteristics, workforce composition, treatment translation, and management of germline findings were assessed. Associations between institutional factors and CGP utilization or trial-related treatment translation were analyzed using incidence rate ratios (IRRs) with 95% confidence intervals.

**Results:**

Among 68 institutions with complete CGP volume data, 6,964 CGP tests were performed, including 922 for gynecologic malignancies. The median number of gynecologic CGP tests per institution was 10. In multivariate analysis, the number of board-certified obstetrician–gynecologists was associated with CGP utilization (IRR, 1.05; 95% CI 1.01–1.08). CGP-guided therapy was delivered to 80 patients (8.7%). Trial-related treatment translation was associated with the number of board-certified obstetrician–gynecologists (IRR, 1.09; 95% CI 1.02–1.16) and the presence of a medical oncology department (IRR, 7.93; 95% CI 1.41–44.72). Presumed germline pathogenic variants were identified in 100 cases (10.8%); however, confirmatory germline testing was performed in only 38 cases.

**Conclusion:**

CGP utilization in Japanese gynecologic oncology was associated with gynecologic workforce capacity and multidisciplinary genomic infrastructure. Collaboration between gynecologic oncology and medical oncology may facilitate treatment translation. CGP may also serve as an entry point for hereditary cancer evaluation despite incomplete downstream germline evaluation.

**Supplementary Information:**

The online version contains supplementary material available at https://doi.org/10.1007/s10147-026-03143-7.

## Introduction

Comprehensive genomic profiling (CGP) has increasingly been incorporated into precision oncology as a platform for identifying genomic alterations with potential therapeutic relevance. High-throughput sequencing technologies allow CGP to simultaneously analyze multiple cancer-related genes, thereby supporting clinical decision-making by improving access to targeted therapies and clinical trials [1, 2]. However, in clinical practice, only a small percentage of patients—typically less than 10%—receive therapies that are matched to their genomic profiles, highlighting the challenges of applying genomic information in treatment [3, 4].

In gynecological oncology, multiple tumor types exhibit molecular heterogeneity, and genomic alterations have been reported across ovarian, endometrial, cervical, and rare gynecological malignancies [5–7]. These molecular features may be relevant for therapeutic stratification or biological characterization; however, the extent to which CGP contributes to clinical decision-making within routine gynecological oncology practice remains unclear.

Most existing evidence on CGP implementation has been generated in Western healthcare systems, where precision oncology programs are often organized around medical oncology–led frameworks and centralized molecular tumor boards [1, 8]. In these settings, genomic testing is often initiated in the context of systemic therapy and is coordinated through multidisciplinary infrastructures. Nevertheless, the applicability of these implementation models to other healthcare systems remains uncertain, particularly in contexts where clinical responsibilities are more heavily distributed across organ-specific specialties.

Japan has developed a nationwide cancer genomic medicine network supported by expert panel systems under a national policy framework [9, 10]. Recent real-world evidence has further demonstrated the clinical utility of CGP in advanced solid tumors within this system [13]. Within this framework, clinical practice in gynecological oncology is frequently led by organ-specific specialists across treatment decision-making and longitudinal disease management. This raises the possibility that the implementation of CGP may follow patterns distinct from those observed in Western models. However, the operationalization of CGP within this system, including institutional variation, workforce involvement, and clinical utilization, has not been systematically evaluated.

Moreover, although tumor genomic profiling may identify indicators of germline alterations, the extent to which these findings are recognized and addressed in standard clinical practice remains insufficiently characterized [11, 12].

To address these gaps, we conducted a nationwide questionnaire survey across institutions involved in Japan’s cancer genomic medicine network. This study aimed to provide a comprehensive overview of the implementation of CGP in gynecological oncology in Japan, focusing on institutional structure, workforce capacity, treatment translation, clinical workflow, and management of germline findings. We also explored institutional factors associated with variations in CGP utilization and trial-related treatment translation.

## Methods

### Study setting and participating institutions

This nationwide questionnaire survey assessed comprehensive genomic profiling (CGP) practices in gynecological oncology in Japan. The questionnaire items are provided in Supplementary Methods S1. The survey targeted 315 institutions accredited as gynecological oncology training facilities by the Japan Society of Gynecologic Oncology (JSGO), including cancer genomic medicine designated core hospitals, designated hospitals, and cooperative hospitals within the national cancer genomic medicine network, as well as non-designated institutions.

Institutional characteristics were collected using two separate classifications. Institution type was self-reported as university hospital, cancer center, or general hospital and was used as an exploratory proxy for institutional function. Cancer genomic medicine designation status was categorized according to the Japanese cancer genomic medicine network as designated core hospital, designated hospital, cooperative hospital, or other facility. In Japan, CGP testing is provided through this government-designated network; designated core hospitals and designated hospitals coordinate expert panel activities, whereas cooperative hospitals conduct CGP testing in collaboration with designated core or designated hospitals. These institutional variables were used to describe the study population and, where appropriate, in sensitivity analyses. The questionnaire was jointly developed by the Japan Society of Obstetrics and Gynecology and JSGO and distributed electronically to designated physicians. Each institution submitted a single consolidated response. Ninety-eight institutions responded (response rate, 31.1%).

Institutional data included annual CGP testing volume, workforce composition, expert panel structure, management of secondary germline findings, referral patterns, and timing of CGP submission. Annual CGP testing volume was defined as the total number of CGP tests performed over a 12-month reporting period, reported as either January–December 2023 or July 2023–June 2024. A cancer genomic medicine unit was defined as an institutional body responsible for coordinating CGP testing and expert panel activities. Because response completeness varied across survey items, the number of institutions included in each analysis differed accordingly. CGP-guided treatment translation was defined as a treatment opportunity or systemic treatment initiated after CGP testing for which treatment selection was based on genomic alterations or biomarkers identified by CGP. This included approved molecularly matched therapies, off-label molecularly targeted therapies, biomarker-selected immune checkpoint inhibitors, clinical trial enrollment, and patient-proposed healthcare services. Standard systemic therapy selected independently of CGP findings was not classified as CGP-guided treatment translation. Treatment categories were reported separately at the institutional level; therefore, overlap between categories could not be excluded.

Because no patient-level data were collected, ethical review and informed consent were waived in accordance with national guidelines.

### Statistical analyses

Annual gynecologic CGP volume was analyzed as a count variable. Given the right-skewed distribution and overdispersion, negative binomial regression models were applied.

Workforce capacity was represented by the number of board-certified obstetrician–gynecologists. Gynecological oncologists were not included separately because they are a subspecialty of obstetrician–gynecologists. Additional variables included the number of clinical geneticists, availability of certified genetic counselors, and involvement of medical oncologists.

Metropolitan classification was defined based on Japan’s three major metropolitan areas: Tokyo (Tokyo, Kanagawa, Saitama, and Chiba), Nagoya (Aichi, Gifu, and Mie), and Osaka (Osaka, Kyoto, and Hyogo). All other prefectures were classified as nonmetropolitan. Trial-related treatment translation was analyzed using the number of trial-related treatments, defined as clinical trial enrollment plus patient-proposed healthcare services, with institutional CGP case volume included as an offset.

Univariate negative binomial regression analyses evaluated associations between workforce variables and annual gynecologic CGP volume. Incidence rate ratios (IRRs) and 95% confidence intervals (CIs) were calculated. All variables were included in multivariate models to assess independent associations. Multicollinearity was assessed using the variance inflation factor, and model fit using the Akaike information criterion. Two-sided *p*-values < 0.05 were considered statistically significant. Analyses were performed using R version 4.3.1.

## Results

### Implementation of comprehensive genomic profiling (CGP) and its association with the gynecological workforce

**Fig. 1:**
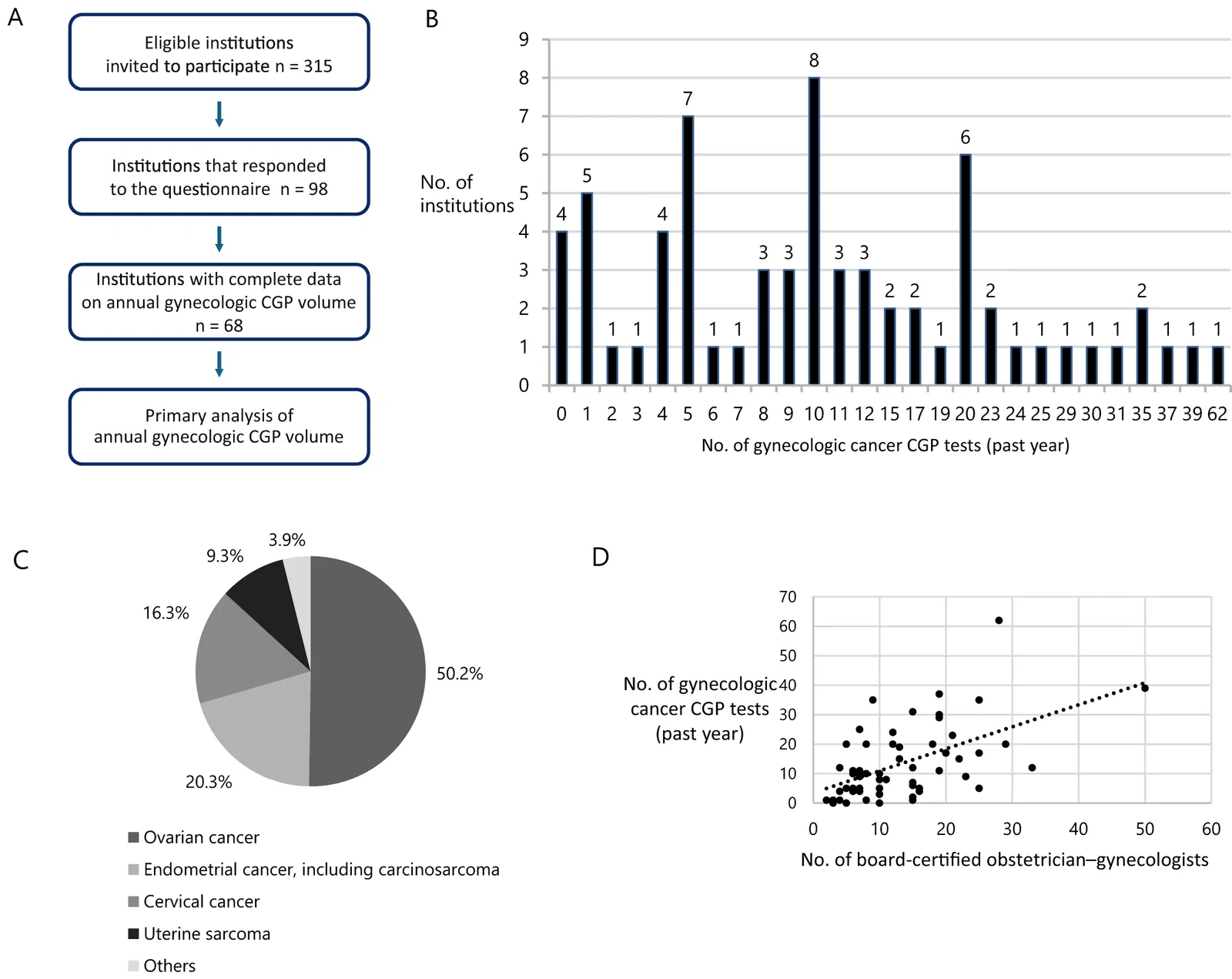
Distribution of gynecological cancer comprehensive genomic profiling (CGP) testing and its association with the gynecological workforce. **A** Flow diagram of participating institutions and analytic cohorts. **B** Distribution of the annual number of gynecological cancer CGP tests per institution among the 68 responding institutions with complete CGP volume data. **C** Distribution of cancer types among 922 gynecological cancer CGP tests reported by these 68 institutions. “Other” cancer types include vaginal cancer, vulvar cancer, gestational trophoblastic disease, and cancer of unknown primary. **D** Association between the number of board-certified obstetrician–gynecologists and the annual number of gynecological cancer CGP tests per institution. Each dot represents one institution

Among the 315 institutions invited to participate, 98 responded to the questionnaire. Complete data for annual gynecologic CGP volume were available from 68 institutions, which constituted the primary analytic cohort for the CGP-volume analysis (Fig. 1A). The institutional characteristics are summarized in Table 1. Of the 98 participating institutions, five were designated core hospitals for cancer genomic medicine (38.5% of 13 nationwide), 18 were designated hospitals (56.3% of 32 nationwide), and 58 were cooperative hospitals (26.5% of 219 nationwide) within the national cancer genomic medicine framework in Japan, whereas 17 institutions (17.3%) were not formally designated and therefore required referral to other designated hospitals. Data on referral-based CGP testing at non-designated institutions are provided in Supplementary Results S1. The mean (median) numbers of board-certified obstetricians–gynecologists and gynecological oncologists per institution were 12.7 (10) and 3.4 (3), respectively (Supplementary Figure S1). A dedicated cancer genomic medicine unit was present in 70 institutions (71.4%). Complete CGP testing data were available from 68 institutions. In these institutions, a total of 6,964 CGP tests were performed, including 922 (13.2%) for gynecological malignancies.

**Table 1:** Characteristics of participating institutions within the national cancer genomic medicine network in Japan

Characteristic	Value	National total
*Institutional designation (Japan cancer genomic medicine framework)*		
Designated Core Hospitals for Cancer Genomic Medicine, n (%)	5 (38.5%)	13
Designated Hospitals for Cancer Genomic Medicine, n (%)	18 (56.3%)	32
Cooperative Hospitals for Cancer Genomic Medicine, n (%)	58 (26.5%)	219
Other facilities, n (% of responding institutions)	17 (17.3%)	
*Gynecologic oncology workforce (per institution)*		
Board-certified obstetricians and gynecologists, mean (median)	12.7 (10)	
Board-certified gynecologic oncologists, mean (median)	3.4 (3)	
*Genomic medicine infrastructure*		
In-house cancer genomic medicine unit present, n (%)	70 (71.4%)	

National total represents the number of institutions designated within the Japanese national cancer genomic medicine network established under the Ministry of Health, Labour and Welfare (MHLW) as of May 1, 2024. Percentages for designated core hospitals, designated hospitals, and cooperative hospitals were calculated using the corresponding national total as the denominator. For “Other facilities”, the percentage was calculated among the 98 responding institutions because this category has no national denominator

The median number of gynecological CGP tests per institution was 10 (mean, 13.0; range, 0–62), with 37 institutions (54.4%) performing ≥ 10 tests annually (Fig. 1B). Among the 922 CGP tests performed for gynecological malignancies, ovarian cancer accounted for the largest proportion (463, 50.2%), followed by endometrial cancer (187, 20.3%), cervical cancer (150, 16.3%), uterine sarcoma (86, 9.3%), and other malignancies (36, 3.9%) (Fig. 1C).

In univariate negative binomial regression analyses, the number of board-certified obstetrician–gynecologists was modestly associated with annual gynecologic CGP volume (IRR, 1.05; 95% CI, 1.02–1.08; *p* = 0.0004). The availability of certified genetic counselors was also associated with annual gynecologic CGP volume (IRR, 2.35; 95% CI 1.18–4.71; *p* = 0.015). (Table 2 and Supplementary Figure S1B). In contrast, neither the number of clinical geneticists (IRR, 1.06; 95% CI 0.97–1.17; *p* = 0.18) nor the presence of medical oncologists (IRR, 1.28; 95% CI 0.75–2.17; *p* = 0.37) showed significant associations.

**Table 2:** Workforce factors associated with annual gynecologic CGP volume

	Univariable IRR		Multivariable IRR	
	(95% CI)	*p*-value	(95% CI)	*p*-value
No.of certified obstetrician–gynecologists	1.05 (1.02–1.08)	0.0004	1.05 (1.01–1.08)	0.005
No.of certified clinical geneticist	1.06 (0.97–1.17)	0.18	1.00 (0.90–1.11)	0.99
Availability of certified genetic counselors	2.35 (1.18–4.71)	0.015	1.70 (0.84–3.44)	0.14
Presence of medical oncology department	1.28 (0.75–2.17)	0.37	1.04 (0.60–1.79)	0.89
*Exploratory alternative workforce model*				
No.of certified gynecologic oncologists	1.19 (1.08–1.31)	0.0004	1.17 (1.06–1.29)	0.002

An exploratory alternative workforce model was constructed by replacing the number of board-certified obstetrician– gynecologists with the number of board-certified gynecologic oncologists. Both variables were not included simultaneously in the primary model because they represent overlapping aspects of gynecologic workforce capacity

In the multivariate model, only the number of board-certified obstetrician–gynecologists remained independently associated with annual gynecologic CGP volume, although the magnitude of the association was modest (IRR, 1.05; 95% CI 1.01–1.08; *p* = 0.005). The effect of certified genetic counselors was attenuated and no longer statistically significant (IRR, 1.70; 95% CI 0.84–3.44; *p* = 0.14), while clinical geneticists (IRR, 1.00; 95% CI 0.90–1.11; *p* = 0.99) and medical oncologists (IRR, 1.04; 95% CI 0.60–1.79; *p* = 0.89) remained non-significant (Table 2). In sensitivity analyses using available proxies for institutional scale and function, hospital bed capacity was not significantly associated with annual gynecologic CGP volume, whereas university hospitals showed higher annual gynecologic CGP volume than general hospitals; cancer centers did not differ significantly from general hospitals (Supplementary Table 1). Consistent with these findings, a positive correlation was observed between the number of board-certified obstetrician–gynecologists and CGP testing volume (Pearson’s *r* = 0.57) (Fig. 1D). As an exploratory normalized analysis, we also examined the proportion of gynecologic CGP tests among all CGP tests to account for overall institutional CGP activity. This proportion was not significantly associated with either the number of board-certified obstetrician–gynecologists or the number of board-certified gynecologic oncologists in Pearson or Spearman correlation analyses (Supplementary Table 2).

### CGP-guided treatment and factors associated with trial-related treatment

**Fig. 2:**
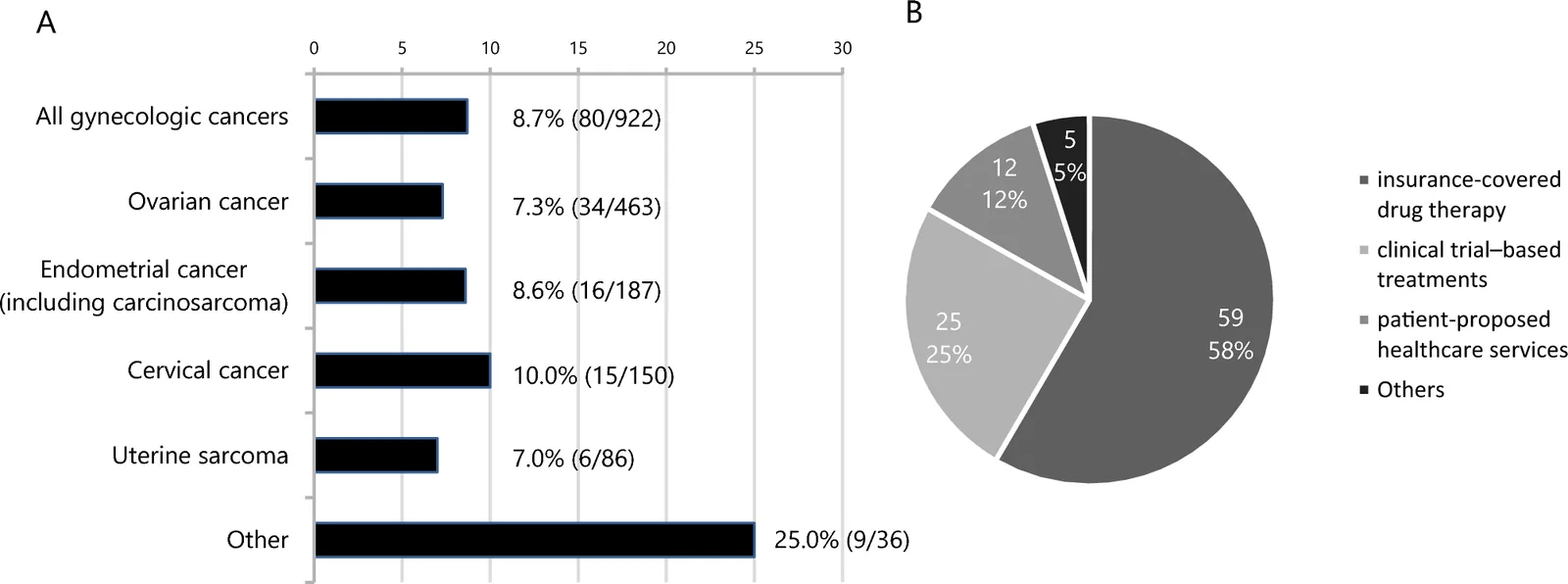
CGP-guided treatment following comprehensive genomic profiling in gynecological malignancies. **A** Proportion of gynecological cancer cases that proceeded to CGP-guided systemic therapy, among the 922 CGP tests reported by the 68 responding institutions with complete CGP volume data. **B** Distribution of CGP-guided treatment modalities, including insurance-covered drug therapy, clinical trial–based treatments, treatments provided under the patient-proposed healthcare services system, and other treatment approaches. Treatment categories were not mutually exclusive

Among the 922 patients with gynecological malignancies who underwent CGP, 80 (8.7%) received CGP-guided systemic therapy. Treatment transition rates by cancer type were 7.3% (34/463) for ovarian cancer, 8.6% (16/187) for endometrial cancer, 10.0% (15/150) for cervical cancer, 7.0% (6/86) for uterine sarcoma, and 25.0% (9/36) for other gynecological malignancies (Fig. 2A).

Treatment modalities included insurance-covered therapies (*n* = 59), clinical trial enrollment (*n* = 25), patient-proposed healthcare services (*n* = 12), and off-label drug use or other treatment approaches (*n* = 5). Because some patients received multiple treatments, 101 treatment instances were reported in total (Fig. 2B). Overall, more than 40% of reported CGP-guided treatment instances were provided outside standard insurance coverage, particularly through clinical trial participation.

To identify institutional factors associated with trial-related treatment translation, we performed negative binomial regression analysis using the number of trial-related treatments, defined as clinical trial enrollment plus patient-proposed healthcare services, relative to institutional CGP case volume. In univariate analysis, the number of board-certified obstetrician–gynecologists (IRR, 1.07; 95% CI 1.02–1.12; *p* = 0.003) and the presence of a medical oncology department (IRR, 4.59; 95% CI 1.31–16.00; *p* = 0.017) were significantly associated with higher trial-related treatment rates, whereas metropolitan classification (other areas vs. three major metropolitan areas) and institutional type (university hospital or cancer center vs. community hospital) were not significantly associated (Table 3). In multivariate analysis, both the number of board-certified obstetrician–gynecologists (IRR, 1.09; 95% CI 1.02–1.16; *p* = 0.014) and the presence of a medical oncology department (IRR, 7.93; 95% CI 1.41–44.72; *p* = 0.019) remained independently associated with higher trial-related treatment rates. In contrast, neither metropolitan classification nor institutional type showed an independent association (Table 3). These findings suggest that trial-related treatment translation after CGP was associated with organ-specific workforce capacity and medical oncology infrastructure rather than geographic location or broad institutional category.

**Table 3:** Institutional factors associated with trial-related treatment translation after CGP

	Univariable IRR (95% CI)	*p*-value	Multivariable IRR (95% CI)	*p*-value
Metro area (Other vs 3 major metropolitan areas)	2.10 (0.88–5.01)	0.096	1.59 (0.56–4.45)	0.382
University hospital/Cancer center (vs general hospital)	2.02 (0.76–5.35)	0.156	0.42 (0.10–1.71)	0.225
No. of board-certified obstetrician–gynecologists (per 1 increase)	1.07 (1.02–1.12)	0.003	1.09 (1.02–1.16)	0.014
Presence of medical oncology department	4.59 (1.31–16.00)	0.017	7.93 (1.41–44.72)	0.019

Trial-related treatments were defined as clinical trial participation plus patient-proposed healthcare services. Negative binomial regression models were used with institutional CGP case volume included as an offset. Values are presented as incidence rate ratios (IRRs) with 95% confidence intervals (CIs). CGP, comprehensive genomic profiling

### Germline findings and clinical integration

**Fig. 3:**
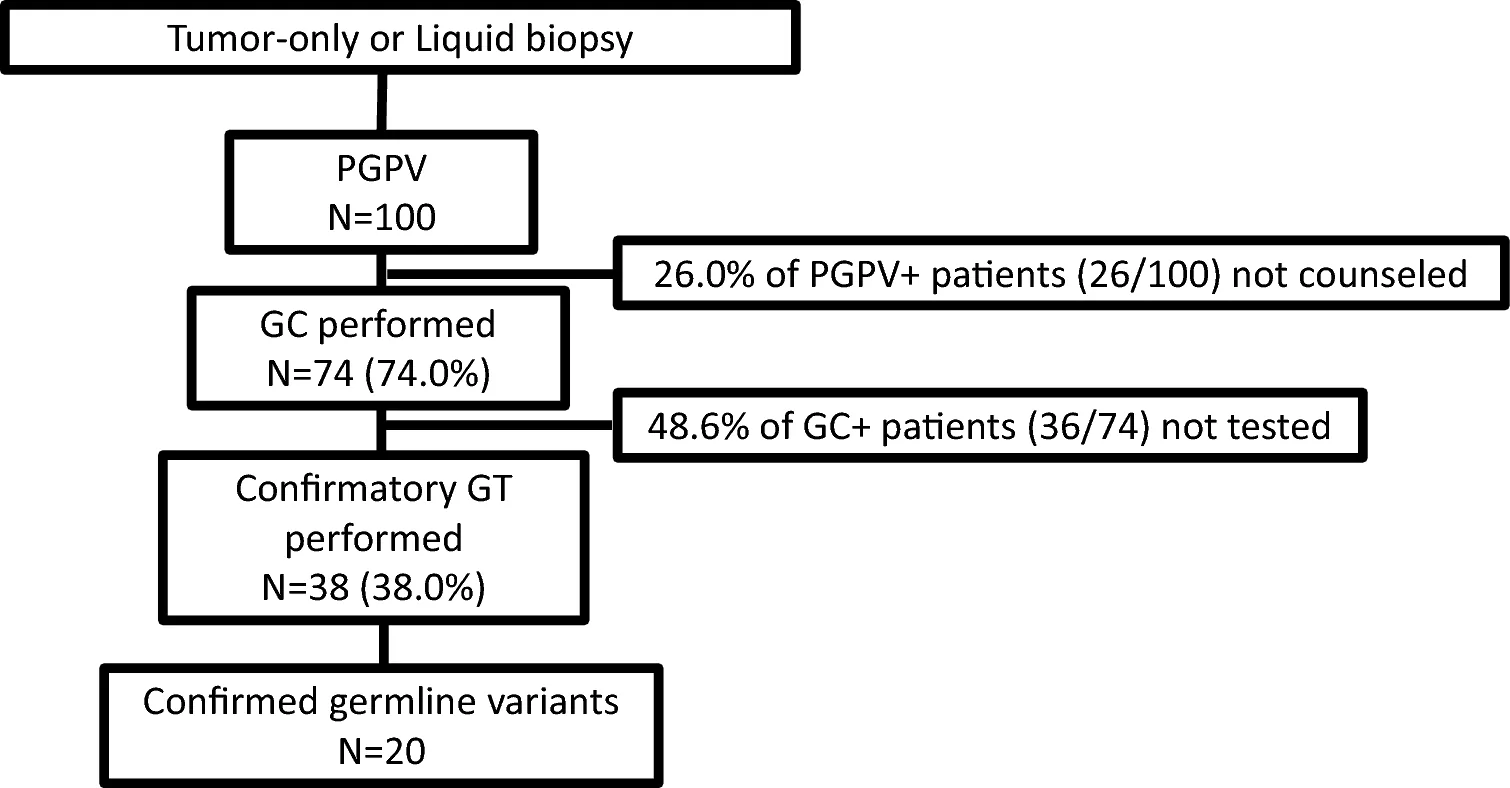
Clinical flow of presumed germline pathogenic variants identified through comprehensive genomic profiling in gynecological malignancies. GC, genetic counseling; GT, germline testing; PGPV, presumed germline pathogenic variant

Among the 922 CGP tests performed for gynecological malignancies, PGPVs were reported in 100 cases (10.8%). Of these, 69 (69.0%) had not previously undergone germline testing, such as BRACAnalysis, indicating that these potential germline findings were first detected through tumor genomic profiling. Genetic counseling was provided in 74 cases (74.0%), and confirmatory germline testing was performed in 38 of these 74 cases (51.4%). Among those tested, 20 of 38 cases (52.6%) were confirmed as germline pathogenic variants. Because confirmatory germline testing was performed in only 38 of 100 PGPV-positive cases, this proportion should be interpreted in the context of incomplete downstream genetic evaluation and does not represent the overall prevalence of germline pathogenic variants among all PGPV-positive cases (Fig. 3). Seven additional GPVs were identified through tumor–normal paired testing, of which four were newly detected without prior germline testing; result disclosure was completed in three cases.

The availability of genetic medicine personnel varied across institutions. Board-certified clinical geneticists were present in 70 institutions (71.4%), and certified genetic counselors were available in 67 institutions (68.4%) (Supplementary Figure S2). Overall, 85% of institutions reported access to genetic counseling services, and gynecologists were involved in genetic counseling in 52 institutions (53.1%). These findings indicate that many institutions had established pathways for managing germline findings, whereas confirmatory testing and result disclosure remained incomplete in a subset of cases.

### Clinical practice structure and physician roles in CGP

Responses regarding clinical workflow were obtained from 92 of 98 institutions, with multiple selections permitted; therefore, percentages are not mutually exclusive. Obstetrician–gynecologists, including treating physicians and those serving as genomic medicine leads, were involved in obtaining informed consent and providing result disclosure in 66 of 92 institutions (71.7%). Medical oncologists within genomic medicine departments were involved in 23 institutions (25.0%), and clinical geneticists in 16 institutions (17.4%).

In expert panel discussions, treating obstetrician–gynecologists participated in 72 of 92 institutions (78.3%). Physicians from genomic medicine departments participated in 47 institutions (51.1%), and obstetrician–gynecologists serving as genomic medicine leads participated in 29 institutions (31.5%).

For disclosure of germline findings identified through CGP, obstetrician–gynecologists were involved in 73 of 92 institutions (79.3%), compared with 19 institutions (20.7%) for physicians from genomic medicine departments without clinical genetics certification and 15 institutions (16.3%) for clinical geneticists. Additional descriptive findings regarding preferred timing of CGP and specimen selection are provided in Supplementary Results S1.

Overall, obstetrician–gynecologists were involved across major components of CGP practice, including informed consent, expert panel discussions, and disclosure of germline findings.

## Discussion

This nationwide questionnaire survey describes patterns of CGP utilization in gynecological oncology among responding institutions in Japan and identifies institutional and workforce factors associated with CGP testing volume and treatment translation. First, annual gynecologic CGP volume was independently associated with the number of board-certified obstetrician–gynecologists. Second, trial-related treatment opportunities after CGP were associated with gynecological workforce capacity and the presence of a medical oncology department, rather than institutional type or geographic classification. Third, CGP functioned not only as a tool for treatment exploration but also as an entry point for hereditary cancer evaluation, although downstream genetic evaluation was not uniformly completed.

A key finding of this study is the association between gynecologic workforce capacity and CGP testing volume. In multivariate analysis, the number of board-certified obstetrician–gynecologists was independently associated with annual gynecologic CGP volume, suggesting that CGP utilization may be linked to the clinical capacity of organ-specific specialists managing gynecological malignancies. This finding should be interpreted as an association rather than evidence of a specific implementation model. Many Western precision oncology programs are organized around medical oncology departments and centralized molecular tumor boards [2, 13], where genomic profiling is frequently initiated as part of systemic therapy decision-making. In the Japanese gynecological oncology setting, obstetrician–gynecologists were involved in CGP consent and result disclosure in most institutions and frequently participated in expert panel discussions. These workflow data suggest that organ-specific clinicians play an important role in CGP-related processes for gynecological malignancies within the national genomic medicine system. However, when we examined the proportion of gynecologic CGP tests among all CGP tests as an exploratory normalized measure relative to overall institutional CGP activity, this proportion was not significantly associated with either the number of board-certified obstetrician–gynecologists or the number of board-certified gynecologic oncologists. Therefore, the association observed in the primary analysis should be interpreted as reflecting annual gynecologic CGP volume rather than a center-volume–independent implementation rate. Residual confounding by overall institutional CGP activity or center volume cannot be excluded.

Recent real-world studies further support the clinical relevance of molecular tumor boards in gynecological oncology. Precision medicine guided by molecular tumor board recommendations has been associated with improved outcomes in cohorts including gynecological cancers, and gynecology-focused molecular tumor board experience has shown that genomic profiling can influence treatment decision-making in patients with gynecological malignancies [14, 15]. Our findings are consistent with this concept and suggest that organ-specialty involvement may also be associated with CGP utilization at the institutional level.

Trial-related treatment translation after CGP was associated with gynecological workforce capacity and the presence of a medical oncology department, rather than metropolitan location or broad institutional category. This finding suggests that treatment access after CGP depends less on institutional labels or geography than on the functional capacity to identify eligible patients, interpret genomic findings in clinical context, and coordinate access to non-standard treatment pathways. In Japan, the Japanese Gynecologic Oncology Group (JGOG) has developed nationwide collaborative efforts to improve clinical trial access for patients with rare and refractory gynecological malignancies, including molecularly guided trials such as CYH33-G201 [16, 17]. These findings are consistent with the view that gynecologic oncologists may contribute not only to CGP-related clinical workflows but also to the proactive identification and referral of patients who may benefit from trial-related treatment opportunities. The association with the presence of a medical oncology department suggests that access to trial-related treatment opportunities after CGP may be facilitated by collaboration with physicians experienced in clinical trials, experimental therapeutics, and patient-proposed healthcare services. However, the confidence interval was wide, indicating substantial uncertainty in the magnitude of this association. Therefore, this finding should be interpreted cautiously, as it may reflect broader institutional clinical trial infrastructure rather than the independent effect of a medical oncology department alone. Despite these favorable structural factors, the overall CGP-guided treatment rate was 8.7%, consistent with real-world precision oncology programs reporting rates of 5–15% [3, 4]. A nationwide C-CAT analysis demonstrated that high-evidence therapeutic alterations are identified in approximately 8% of patients [10], and recent real-world data support the clinical utility of CGP in advanced solid tumors in Japan [18]. These findings indicate that increasing annual CGP testing volume alone may not be sufficient to improve therapeutic yield; timely testing, trial availability, eligibility assessment, and interdisciplinary treatment coordination are also required to convert genomic information into actionable treatment. Because travel burden can limit participation in genotype-matched trials after CGP, even nationwide referral networks may not fully overcome geographic barriers [19]. Broader adoption of decentralized trial approaches may therefore be important to reduce travel-related burdens and expand equitable access to genomically matched therapies [20].

Beyond therapeutic decision-making, CGP also contributed to identifying potential hereditary cancer risks. PGPVs were identified in 10.8% of gynecological CGP cases, many in patients without prior germline testing. These findings support the role of CGP as a platform that can inform both treatment exploration and hereditary cancer evaluation. Tumor genomic profiling frequently identifies germline pathogenic variants [4, 5], and this function is particularly relevant in gynecological malignancies, in which hereditary cancer syndromes account for a clinically important subset of cases [6]. However, downstream genetic evaluation was not uniformly implemented. Although genetic counseling was provided in most PGPV-positive patients, confirmatory germline testing was performed in only 38 of 100 PGPV-positive cases. Therefore, the proportion of confirmed germline pathogenic variants should be interpreted cautiously and does not represent the overall prevalence of germline pathogenic variants among all PGPV-positive cases. Disclosure of germline findings identified through tumor–normal paired testing was also incomplete in some cases. These findings highlight the challenge of integrating tumor genomic profiling into structured hereditary cancer care pathways. As obstetrician–gynecologists were frequently involved in disclosure of germline-related findings, strengthening collaboration among organ-specific clinicians, genetic counselors, clinical geneticists, and genomic medicine units may be important to maximize the clinical utility of CGP-derived germline findings.

The role of tumor–normal paired CGP may become increasingly important in this setting. Paired tumor–normal analysis can distinguish germline from somatic variants, and nationwide GenMineTOP data have provided insight into germline pathogenic variants detected through paired CGP in Japan [21]. Since GenMineTOP, a dual DNA–RNA CGP test using paired tumor–normal samples, was reimbursed in Japan in August 2023 [22], gynecologists may increasingly need to consider both therapeutic opportunities and the management of germline findings when selecting an appropriate CGP platform.

This study has some limitations. First, it was based on questionnaire responses and may be subject to reporting bias. Although the questionnaire was distributed to 315 eligible institutions, 98 institutions responded, and complete CGP-volume data were available from 68 institutions for the primary analysis. Institutions with greater activity in cancer genomic medicine, greater institutional resources, or higher CGP testing volume may have been more likely to respond and to provide complete data. Therefore, the findings from the CGP-volume analysis may not fully represent all eligible institutions in Japan. Second, detailed patient-level data, including genomic alterations, treatment eligibility, clinical outcomes, and other clinical factors, were not evaluated because this study used institution-level aggregated data. Finally, the cross-sectional design precludes causal interpretation of the associations between workforce capacity, institutional infrastructure and annual gynecologic CGP volume or treatment translation.

In conclusion, CGP utilization in Japanese gynecological oncology was associated with gynecologic workforce capacity and multidisciplinary genomic infrastructure. Although overall treatment rates remain modest, translating CGP findings into treatment requires collaboration between gynecological oncology and medical oncology. CGP may also serve as an entry point for hereditary cancer evaluation; however, incomplete downstream germline evaluation highlights the need for structured pathways linking tumor genomic profiling with genetic counseling and confirmatory germline testing.

## Supplementary Information

Below is the link to the electronic supplementary material.

Supplementary Material 1

## Data Availability

The datasets generated and/or analyzed during the current study are not publicly available because they contain information that could compromise research participant privacy but are available from the corresponding author on reasonable request.
